# Revealing very small FLT3 ITD mutated clones by ultra-deep sequencing analysis has important clinical implications in AML patients

**DOI:** 10.18632/oncotarget.5161

**Published:** 2015-09-05

**Authors:** Elisa Zuffa, Eugenia Franchini, Cristina Papayannidis, Carmen Baldazzi, Giorgia Simonetti, Nicoletta Testoni, Maria Chiara Abbenante, Stefania Paolini, Chiara Sartor, Sarah Parisi, Giovanni Marconi, Federica Cattina, Maria Teresa Bochicchio, Claudia Venturi, Emanuela Ottaviani, Michele Cavo, Giovanni Martinelli

**Affiliations:** ^1^ “Seràgnoli” Institute of Hematology, Sant'Orsola-Malpighi University Hospital, Bologna, Italy; ^2^ Bone Marrow Transplant Unit, University of Brescia, Brescia, Italy

**Keywords:** AML, FLT3, ultra-deep sequencing, clonal evolution, minimal residual disease

## Abstract

*FLT3* internal tandem duplication (ITD), one of the most frequent mutations in Acute Myeloid Leukemia (AML), is reported to be an unstable marker, as it can evolve from *FLT3* ITD- to ITD+ during the disease course. A single-gene sensitive mutational screening approach may be helpful for better clarifying the exact timing of mutation occurrence, especially when *FLT3* ITD appears to occur late, at disease progression. We developed an amplicon-based ultra-deep-sequencing (UDS) approach for *FLT3* mutational screening. We exploited this highly sensitive technology for the retrospective screening of diagnosis, relapse and follow-up samples of 5 out of 256 cytogenetically normal (CN-) AML who were *FLT3* wild-type at presentation, but tested ITD+ at relapse or disease progression. Our study revealed that all patients carried a small ITD+ clone at diagnosis, which was undetectable by routine analysis (0,2–2% abundance). The dynamics of ITD+ clones from diagnosis to disease progression, assessed by UDS, reflected clonal evolution under treatment pressure. UDS appears as a valuable tool for *FLT3* mutational screening and for the assessment of minimal residual disease (MRD) during follow-up, by detecting small ITD+ clones that may survive chemotherapy, evolve over time and definitely worsen the prognosis of CN-AML patients.

## INTRODUCTION

Mutations in the FMS-related tyrosine kinase 3 (*FLT3*) gene represent one of the most frequent and clinically challenging event in Acute Myeloid Leukemia (AML), occurring in the 25% of newly diagnosed cases [1]. Two types of FLT3 mutations have been described: activating internal tandem duplications (*FLT3* ITD) in or near the juxtamembrane domain of the receptor, and point mutations within the activation loop of the tyrosine kinase domain (*FLT3* TKD) [2]. *FLT3* ITD are independent predictors of bad prognosis in cytogenetically normal (CN-) AML and they represent a challenge of great interest, due to the high frequency and to the availability of specific inhibitors [3–5].

In the absence of *FLT3* ITD, Nucleophosmin 1 (*NPM1*) mutations generally confer a favourable prognosis and NPM1-mutated AML cases do not undergo allogenic transplantation in first complete remission (CR1). High *FLT3* ITD allelic burden (greater than 33%) instead associates with poor prognosis and high relapse rate (RR). Therefore, high level mutant *FLT3* ITD patients are candidate for transplantation in CR1, according to donor availability [6, 7]. Moreover, recent studies showed that also low levels of *FLT3* ITD (defined between 5% and 25% of mutant level) predict an increased cumulative incidence of relapse (CIR), independently of *NPM1* mutational status. Since the RR is among the most relevant parameters guiding decisions about allogenic transplantation in CR1, also low *FLT3* ITD levels should be taken into account [8, 9].

Several studies reported that *FLT3* ITD is an unstable marker during disease evolution, with a non-homogeneous allelic burden and with cases progressing from *FLT3* ITD- to *FLT3* ITD+ status and viceversa [10, 11]. Recent whole genome sequencing analyses provided new insights into the origin and clonal evolution of AML mutations: “initiating” driver mutations (as *FLT3* ITD) are early events and are relevant to leukemogenesis and targeted therapy, while cooperating mutations occur later and contribute to disease progression [12]. This evidence suggests that a single-gene ultra-deep mutational screening approach may be helpful to monitor *FLT3* mutational status over time and to promptly identify *FLT3* ITD clones expanding at relapse or disease progression [13].

Next-generation sequencing (NGS) has overcome the technical limits of routine molecular diagnostics methods. The high sensitivity of NGS technologies allows discriminate between polyclonal and compound mutations and to accurately quantify the mutated clone abundance [14–16]. Therefore, NGS appears as an attractive strategy to detect *FLT3* mutations in AML patients, especially in cases showing a complex pattern of clonal evolution, in which *FLT3* mutations evolve during the disease course.

We aimed to understand the dynamics and evolution of ITD mutations in CN-AML cases, which showed *FLT3* ITD positivity only at follow-up by conventional sequencing methodologies. To this purpose, we moved from a PCR assay followed by denaturing HPLC analysis (1–5% sensitivity) to an amplicon-based ultra-deep-sequencing (UDS) approach, which is currently the most sensitive approach, by exploiting the Roche 454 Life Sciences technology [17, 18]. We analyzed diagnosis, relapse and follow-up samples of 5 out of 256 CN-AML patients, who tested negative for *FLT3* ITD at diagnosis by routine approaches, but carried *FLT3* ITD at relapse or disease progression. Retrospective UDS screening of *FLT3* mutational status in these patients revealed the presence of small ITD+ clones, which were undetectable by routine analysis.

## RESULTS

### Robustness of UDS for *FLT3* mutational analysis

#### Sequencing run metrics

Sequencing runs generated an average of 121122 reads (range, 69624–171904). Sequencing depth was between 1951 and 14273 reads per base across the runs (average 8077).

#### Sensitivity of mutation detection

To test the method sensitivity in the detection of mutated clones, we took advantage of the AML cell lines MOLM-13 (harbouring a heterozygous ITD mutation) and OCI-AML3 (expressing wild-type *FLT3*). We diluted MOLM-13 RNA with OCI AML3 RNA, thus obtaining the following rates of *FLT3* ITD mutation: 50% (undiluted), 25% (1:2 dilution), 5% (1:10 dilution), 2,5% (1:20 dilution), 0,25% (1:200 dilution), 0,125% (1:400 dilution) and 0,06% (1:800 dilution).

**Figure 1:**
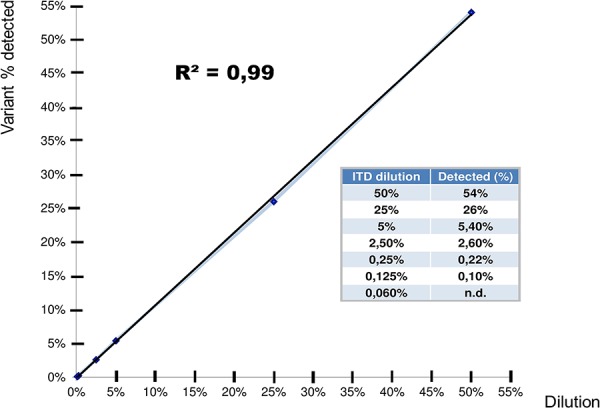
Sensitivity of mutation detection Sensitivity of mutation detection was measured by serial dilutions of MOLM-13 with OCI-AML3 RNA (harbouring a heterozygous ITD mutation ad wild-type *FLT3*, respectively). The following dilutions of MOLM-13 RNA were tested: 50% (undiluted), 25% (1:2 dilution), 5% (1:10 dilution), 2,5% (1:20 dilution) and 0,25% (1:200 dilution), 0,125% (1:400 dilution) and 0,06% (1:800 dilution).

Sequencing results showed signals of mutation down to 0,125% mutation abundance, which we consider the detection limit in our experimental setting. The concordance between the average variant frequencies detected at different dilution levels and the expected frequencies are shown in Figure 1.

#### Inter- and intra-run reproducibility

Inter-run reproducibility of UDS was assessed by sequencing MOLM-13 samples obtained by separate RT and PCR steps in two independent runs. Intra-run reproducibility was tested by sequencing 3 replicate MOLM-13 samples with 3 different barcode indexes in the same run. The samples showed comparable ITD variant frequency, thus confirming high inter- and intra-assay reproducibility of the results (Table 1).

**Table 1:** Inter-run and intra-run reproducibility

		RUN1			RUN2					
		REPL.1	REPL.2	REPL.3	REPL.1	REPL.2	REPL.3	MEAN	SD	CV
MOLM-13	ITD (50%)	53,92	55,57	53,38	53,16	50,99	53,18	53,37	1,47	0,03

Inter- and intra-run reproducibility were tested in repeated runs using independent MOLM-13 replicates (including independent RT and PCR steps between run1 and 2).

#### Concordance between conventional and UDS methods in clinical sample testing

To verify the performance of UDS in the detection of ITD mutations in clinical AML samples, we performed parallel UDS and conventional analyses on 20 AML samples from patients enrolled in an experimental clinical trial (AMBIT FLT3 inhibitor AC220–002). ITD mutation analysis was performed before and after treatment, by PCR and D-HPLC followed by Sanger sequencing and by UDS. The results are shown in Table 2: UDS detected all the ITD mutations identified by conventional analysis, with 100% concordance in terms of ITD size and sequence. Moreover, 2 ITD mutations that were under the detection limit of conventional tests could be detected by UDS (underlined in red in Table 2). The results confirmed the concordance between conventional and UDS methods in clinical sample testing and highlighted the highest sensitivity of UDS.

**Table 2:** Concordance between conventional and UDS methods in clinical sample testing

			Baseline		Post AC220	
UPN	Age	Cytogenetic Profile	BMB (%)	FLT3 ITD (%)	BMB (%)	FLT3 ITD (%)
**6**	63	46XY	100	**ITD 1863 (33bp):0,7%** **ITD 1906 (36bp):77%**	100	**ITD 1906(36bp):96%**
**7**	71	46XY	100	**ITD 1920 (90bp):29%**	5	**ITD 1920 (90bp):5%**
**8**	40	46XX	80	**ITD 1922(69 bp): 1,8%**	100	**ND**
**9**	55	46XX;t(2;8)(q15,q24)	100	**ITD 1874 (45bp):39,8%**	100	**ITD 1874 (45bp):54,4%**
**10**	60	46XY	80	**ND**	90	**ND**
**11**	63	Complex	100	**ND**	100	**ND**
**12**	70	47XY;+11	90	**ITD 1909 (45bp):27,3%**	80	**ITD 1909 (45bp):20,7%**
**13**	50	46XX;t(6,11)(q27,q23)	100	**ND**	100	**ND**
**14**	55	47XX;+21	100	**ND**	100	**ND**
**15**	67	46XX	80	**ND**	80	**ND**

Clinical AML samples from patients treated with the *FLT3* inhibitor AC220 were screened for the presence of ITD mutations before and after therapy by PCR, followed by DHPLC and Sanger Sequencing, and by UDS (BMB: bone marrow blasts, ND: not detected). The ITD mutations in red were detected exclusively by UDS analysis.

#### UDS revealed multiple minimal ITD mutated clones present at diagnosis and undiscovered by Sanger-based assay

To explore the clonal evolution of *FLT3* mutations, 22 AML samples collected from 5 patients were analyzed by UDS (three or more time points per patient).

**Figure 2:**
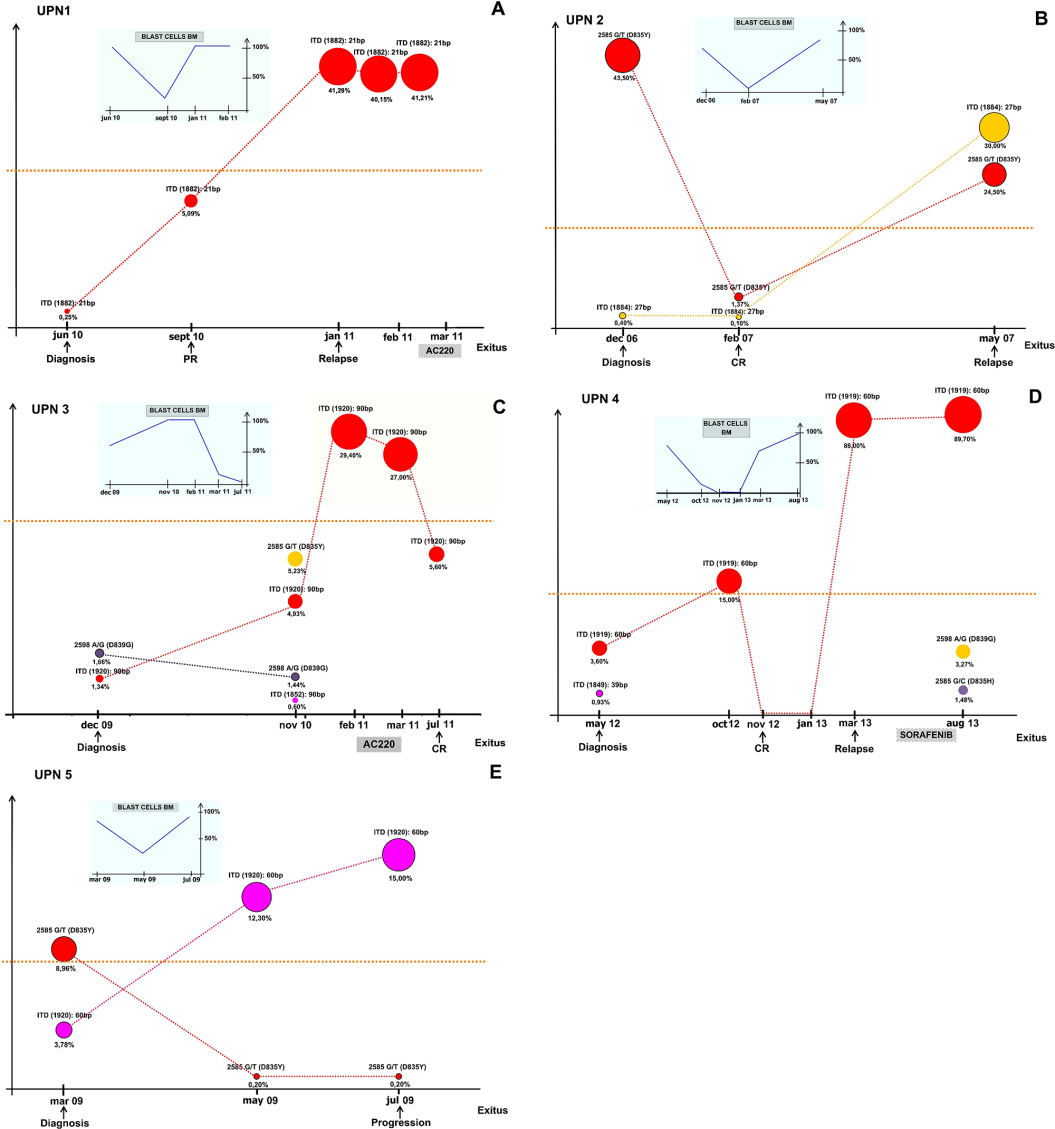
Evolution of *FLT3* ITD+ clones during the disease course in 5 CN-AML analysed by UDS Circles dimensions represent the percentage of the ITD+ clones over total *FLT3* RNA during follow-up; colours indicate mutated clones and the dotted lines mark the threshold of detection of routine Sanger Sequencing method. For each patient, the *FLT3* mutational status and clone abundance is reported at each time point of analysis. The small graph shows the percentage of bone marrow blast cells all over the disease course. (PR: partial remission; CR: complete remission; BM: bone marrow).

The mutations detected by conventional analysis were confirmed by UDS (relapse and disease progression samples of 3 and 2 patients, respectively). Moreover, 8 samples showed one or more additional *FLT3* ITD+ clone by UDS, which were not detectable by routine analysis (dots below the dotted line in Figure 2). Notably, all ITD+ clones becoming detectable at follow up by routine analysis were found at low frequency (0,2% to 2%) at the time of diagnosis by UDS. Four cases (UPN 1, 2, 3 and 5) carried a single ITD mutation, while UPN 4 had two small ITD mutated clones and one of them expanded over time (ITD (1919), Figure 2).

#### Clonal evolution of *FLT3* ITD mutations assessed by UDS analysis

Patients UPN 1 and UPN 3, who were not treated with conventional chemotherapy at diagnosis, showed a progressive expansion of the ITD+ clone at follow-up (Figure 2A and 2C). UPN1 had a small ITD+ clone at diagnosis (0,25% by UDS). Despite obtaining an initial partial remission by cytoreduction with Hydroxyurea, the patient relapsed seven months after diagnosis, due to the progressive expansion of the ITD+ clone which became the dominant one (41,29%) and remained stable over time (Figure 2A).

Patient UPN 3 received best supportive therapy (BST). The ITD+ clone progressively increased (from 1,34% at diagnosis to 29,4% after 14 months of follow-up), along with the appearance of a minor ITD+ clone (0,6%) and two small TKD mutated clones (D835Y and D839G at 5,23% and 1,44% of abundance, respectively) in the sample tested 11 months after diagnosis. The patient was enrolled in an experimental clinical trial (2 cycles of AMBIT FLT3 inhibitor AC220–002 oral compound, at the dosage of 135 mg/daily for 28-days cycles) and achieved a complete morphological remission, along with the decrease of the major *FLT3* ITD mutated clone in the sample tested 5 months after therapy (~5%) (Figure 2C).

Patient UPN 4 received conventional induction chemotherapy and after an initial expansion of the *FLT3* ITD+ clone, he achieved a complete morphological remission at the end of a “3+7” induction schedule. At molecular level, the remission was marked by reduction of the ITD+ clone to undetectable levels by Sanger assay and UDS. The patient then received three courses of consolidation, followed by autologous stem cells apheresis, in preparation for autologous stem cell transplantation. Unfortunately, the patient relapsed four months after achieving remission, when one of the ITD mutated clones, detectable at diagnosis at low levels (3,6%), became homozygous (88%) likely by loss of heterozygosity (LOH) of the mutated allele. LOH was previously reported to be frequent in AML patients, suggesting a general mechanism of AML clonal evolution and disease progression [19]. The patient was treated with the oral tyrosine-kinase inhibitor (TKI) Sorafenib, at the standard dosage of 400 mg twice daily, for one month. The drug was well tolerated, and no adverse events occurred. Unfortunately, no haematological response was observed. Accordingly, after one month of treatment, UDS revealed the persistence of the major ITD+ clone, that remained stable at 89,7%, and the appearance of two additional *FLT3* TKD mutated clones, D839G and D835H at 1,48% and 3,27% of abundance, respectively (Figure 2D). These mutations are able to confer resistance to Sorafenib treatment, as previously reported [20, 21].

Patient UPN 5 showed resistance to conventional induction chemotherapy (Cytarabine and Idarubicine). UDS analysis revealed a progressive expansion of the ITD+ clone over time (from 3,78% at diagnosis to 12,3% two months later), while the TKD mutated clone D835Y was successfully inhibited by the treatment (from 8,96% at diagnosis to 0,2%). The sample tested by UDS at disease progression (4 months after diagnosis) showed a further increase of the ITD mutated clone (15% of abundance), while the D835Y clone remained stable (0,2%).

Finally, patient UPN 2 was characterized by TKD D835Y mutation (43%) and a small ITD mutated clone (revealed only by UDS analysis, 0,4%) at diagnosis. After two months of conventional chemotherapy treatment (3+7 schedule with Gemtuzumab-Ozogamicin, Cytarabine and Idarubicine), the patient obtained a complete morphological remission, with the ITD+ clone remaining stable (~0,1%) and the TKD D835Y mutated clone dropping down (from 43,5% at diagnosis to 1,37% at CR). At relapse, which occurred three months later, UDS analysis revealed that the ITD and D835Y clones increased to 30% and 24,5%, respectively, showing that the extensive use of UDS, associated with correct time-points of molecular monitoring, could be critical for relapse prediction and therapeutic decisions (Figure 2B).

## DISCUSSION

*FLT3* ITD mutations are independent predictors of poor prognosis in AML, particularly in CN-AML patients. They are one of the most frequent and clinically challenging AML aberrations and therapeutic decisions are extensively affected by results and interpretation of *FLT3* mutational screening [6, 22, 23].

Several studies described the instability of *FLT3* ITD during the disease course, with cases evolving from *FLT3* ITD- to FLT3 ITD+ and viceversa [10, 11]. As for the dynamics of clonal evolution, recent whole genome sequencing analyses revealed that AML relapse frequently occurs by outgrowth of sub-clones gaining a small cluster of new mutations, favoured by DNA damage induced by cytotoxic chemotherapy [24]. Among recurrent AML mutations, *FLT3* ITD has been defined a driver mutation, which sustains the founding clone for progression to a frank leukemia [12, 24].

Based on this evidence, we aimed to gain new insights into the dynamics of *FLT3* ITD clones, including outgrowth and clonal evolution in treated CN-AML cases evolved from FLT3 ITD- to *FLT3* ITD+. In those cases, *FLT3* ITD does not appear to be a driver mutation, as it was acquired at disease progression. In our cohort of 886 clinically and molecularly characterized AML patients, who were treated and followed at Seràgnoli Institute of Hematology-University of Bologna between 2002 and 2013, we found 5 CN-AML who were *FLT3* ITD- at diagnosis and became *FLT3* ITD+ at relapse or during follow-up.

Here we provide a high-coverage UDS strategy for *FLT3* mutational testing that is able to detect small ITD mutated clones, even lower than 1% of abundance.

UDS analysis of *FLT3* in the 5 CN-AML from diagnosis to relapse or disease follow-up revealed that all patients actually carried a small ITD+ clone at diagnosis, that was undetectable by conventional Sanger-based analysis (relative abundance of 0,2–2%).

A recent study by *Ottone et al*. exploited a patient-specific real-time quantitative-PCR (RQ-PCR) strategy to increase *FLT3* ITD detection sensitivity of the routine RT-PCR assay [25]. They also observed that patients who were *FLT3* ITD- at diagnosis and relapsed as *FLT3* ITD+, actually had a small original *FLT3* ITD+ clone, which remained undetectable by routine analysis. However, this strategy of ITD detection has a low applicability potential in the routine practice and especially at diagnosis, since it requires a patient-specific RQ-PCR approach. Our method, based on a universal UDS protocol for *FLT3* analysis, is instead applicable on a routine base in the AML molecular diagnostic setting, leading to a great advance in the baseline screening of *FLT3* mutational status in terms of sensitivity.

Since recent studies demonstrated that detection of low allele burden of *FLT3* ITD affects the RR, sensitive methods able to detect even small *FLT3* ITD+ clones become crucial in the routine molecular diagnostics. Indeed, allogeneic transplantation in CR1 should be recommended to all patients showing *FLT3* ITD positivity, irrespective of the allele burden [9]. Accordingly, early detection of *FLT3* ITD positivity of UPN4 case since diagnosis would have argued for alternative therapeutic strategies, including allogeneic stem cell transplantation [26].

Thanks to the high sensitivity in revealing low abundance mutations, UDS could be a valuable tool also for MRD assessment and prediction of clinical resistance to TKI treatment [16, 17, 27].

Its application during disease follow-up could help identify alternative and personalized therapies, based on the patient's molecular status. Clonal evolution analysis by UDS showed that the ITD+ clone became undetectable in one of the two patients who achieved clinical remission after receiving conventional chemotherapy. The other patient had a therapy-resistant ITD+ clone, which remained detectable by UDS also at complete morphological remission (0,1%), suggesting that the high sensitivity of the approach may be a suitable tool for MRD monitoring at follow-up.

The dynamics of therapy-related clonal evolution indicate that small *FLT3* ITD+ subclones may have been selected under chemotherapy pressure and may have expanded, becoming dominant at relapse. Indeed, *FLT3* ITD has been associated with chemoresistance in leukemic cells and the introduction of TKI in the clinical practice has, at least in part, overcome the barrier for eradicating these leukemic clones [24, 28, 29]. In the patient who did not receive conventional chemotherapy, the *FLT3* ITD+ clone progressively expanded, thanks to the proliferative and survival advantage provided by class I AML mutations to hematopoietic progenitors [30].

The ability to detect small mutated clones at early disease stages will be highly relevant to guide therapeutic decisions, especially in *FLT3* ITD+ cases. Indeed, selective TKI treatment can help eradicate the ITD mutated clones in combination with conventional chemotherapy [21]. Moreover, since TKI treatment pressure can induce the selection of mutated resistant clones, UDS analysis will provide early detection of secondary TKD alterations that may impair sensitivity to TKI [20]. This issue is relevant to *FLT3* TKI treatment. Since sequential therapy with *FLT3* inhibitors with diverse resistance profiles may provide clinical benefits, the application of a sensitive method to detect, monitor and act on drug-resistant clones during treatment may be a suitable strategy towards personalized therapeutic approaches [31].

Different NGS strategies and platforms have recently become available and suitable for advanced molecular diagnostics of haematological malignancies. The MiSeq (Illumina) and Ion Proton System (Ion Torrent, Life Technologies) platforms allow high coverage analyses (up to 25 M reads per run for MiSeq and 60–80 M reads for Ion Proton) of a gene panel in multiple samples per run, thus facilitating the study of leukemogenesis and relapse. The Roche GS Junior (454-Life Sciences) System, which generates 100 thousand reads per run, is suitable for testing a single mutated gene in a few patients or few genes in a single sample at diagnosis/relapse or during follow-up for MRD detection.

As the performance of NGS platforms is constantly improving, each technology will be exploited in the near future to answer specific diagnostic questions, thus maximizing cost-effectiveness of molecular analyses in the haematological field [32].

In conclusion, our study clearly indicates that UDS is a valuable tool to reveal small *FLT3* ITD mutated clones that may evolve over time and worsen the prognosis of otherwise good prognosis CN-AML patients, and to optimize therapeutic strategies. UDS of single or few genes will help clarify the dynamics of clonal evolution from diagnosis to relapse and will support whole genome and whole exome sequencing approaches in the understanding of the comprehensive AML clonal architecture.

## MATERIALS AND METHODS

### Patients

We retrospectively reviewed clinical and molecular data of 886 AML patients treated at Seràgnoli Institute of Hematology-University of Bologna between 2002 and 2013: 239 patients were *FLT3* mutated (27%) and, among them, 157 were ITD positive (18%); 256 patients had CN-AML, with 46 being FLT3 ITD mutated (18%). In this group of CN-AML, 5 patients were diagnosed as *FLT3* ITD negative, but at early relapse or during follow-up, the FLT3 mutational status changed and they gained an ITD mutation.

Two of the 5 patients were also *NPM1* mutated at diagnosis. Written informed consent for clinical trial enrolment and biological sample collection was obtained, in accordance with the Declaration of Helsinki. Patients' clinical and biological characteristics are described in Table 3.

**Table 3:** Clinical and biological characteristics of the 5 CN-AML patients analyzed by UDS for *FLT3* mutational status

UPN	Age	Sex	WBC Diagnosis (x10^9^/1)	% Blast (BM)	Diagnosis Date	Karyotype	NPM Diagnosis	FLT3 ITD Diagnosis *	Relapse/Progression Date	Outcome
1	77	M	2200	35	June 2010	46,XY	wild-type	wild-type	Jan 2011	Dead
2	72	F	244000	100	Dec 2006	46,XX	wild-type	wild-type	May 2007	Dead
3	70	M	25700	100	Dec 2009	46,XY	mutant	wild-type	Sept 2011	Dead
4	48	F	11300	100	May 2012	46,XY	mutant	wild-type	Mar 2013	Dead
5	64	M	44000	100	Mar 2009	46,XY	wild-type	wild-type	Jul 2009	Dead

* Determined by routine molecular analysis (PCR and D-HPLC analysis followed by Sanger Sequencing)

(WBC: white blood cells; BM: bone marrow)

### Routine molecular and cytogenetic characterization

Total RNA was extracted from Ficoll-Hypaque isolated bone marrow mononuclear cells collected at diagnosis and during clinical course and converted into c-DNA. Analysis of *FLT3* and *NPM1* gene mutations was performed as previously described [18].

Chromosome banding analysis was performed on bone marrow cells after short-term culture (24 and/or 48 hours). The cells were treated with colchicine and hypotonic solution, the pellet was then fixed and washed in methanol/acetic acid (3:1). The cells were re-suspended in fixative and dropped on slides. Karyotypes were analyzed after G banding and defined according to International System for Human Cytogenetic Nomenclature (ISCN 2013) [33]. Twenty or more metaphases per sample were analyzed.

### Ultra Deep Sequencing of the *FLT3* gene

RNA was converted into cDNA by Transcriptor High-Fidelity cDNA Synthesis kit (Roche Applied Science). The first amplification step was performed by polymerase chain reaction (PCR) using the FastStart High-Fidelity PCR System kit (Roche Applied Science), in order to generate 5 partially overlapping amplicons covering exons 11–24 of the gene. Forward and reverse primers consisted in an adapter sequence for emulsion PCR, a sample specific barcode sequence (multiplex identifier) for sample pooling and the gene-specific sequence. Primer sequences are listed in Table 4. Sequencing was performed on a Roche GS Junior (454-Life Sciences) according to manufacturer's instructions. Data obtained from the sequencing runs were analyzed by Amplicon Variant Analyzer Software (454-Life Sciences), using the reference gene FLT3 (GenBank accession no NM_004119).

**Table 4:** Primers used for UDS analysis

AMPLICON	MID	SEQUENCE 5′ to 3′
1	1	FOR 5′- CGTATCGCCTCCCTCGCGCCATCAGACGAGTGC GTTTGGACCTGGAAGAAGTGTTCA -3′
		REV 5′- CTATGCGCCTTGCCAGCCCGCTCAGACGAGTGCGTCGGTCACCTGTACCATCTGTAG -3′
	2	FOR 5′- CGTATCGCCTCCCTCGCGCCATCAGACGCTCGACATTGGACCTGGAAGAAGTGTTCA -3′
		REV 5′- CTATGCGCCTTGCCAGCCCGCTCAGACGCTCGACACGGTCACCTGTACCATCTGTAG -3′
	3	FOR 5′- CGTATCGCCTCCCTCGCGCCATCAGAGACGCACTCTTGGACCTGGAAGAAGTGTTCA -3′
		REV 5′- CTATGCGCCTTGCCAGCCCGCTCAGAGACGCACTCCGGTCACCTGTACCATCTGTAG -3′
2	1	FOR 5′- CGTATCGCCTCCCTCGCGCCATCAGACGAGTGCGTTTTAACCCTGCTAATTTGTCAC -3′
		REV 5′- CTATGCGCCTTGCCAGCCCGCTCAGACGAGTGCGTATGAGTGCCTCTCTTTCAGA -3′
	2	FOR 5′- CGTATCGCCTCCCTCGCGCCATCAGACGCTCGACATTTAACCCTGCTAATTTGTCAC -3′
		REV 5′- CTATGCGCCTTGCCAGCCCGCTCAGACGCTCGACAATGAGTGCCTCTCTTTCAGA -3′
	4	FOR 5′- CGTATCGCCTCCCTCGCGCCATCAGAGCACTGTAGTTTAACCCTGCTAATTTGTCAC -3′
		REV 5′- CTATGCGCCTTGCCAGCCCGCTCAGAGCACTGTAGATGAGTGCCTCTCTTTCAGA -3′
3	1	FOR 5′- CGTATCGCCTCCCTCGCGCCATCAGACGAGTGCGTAATCCAGGTTGCCGTCAA -3′
		REV 5′- CTATGCGCCTTGCCAGCCCGCTCAGACGAGTGCGTATTCAATTTCATCTTCAGAGTGA -3′
	2	FOR 5′- CGTATCGCCTCCCTCGCGCCATCAGACGCTCGACAAATCCAGGTTGCCGTCAA -3′
		REV 5′- CTATGCGCCTTGCCAGCCCGCTCAGACGCTCGACAATTCAATTTCATCTTCAGAGTGA -3′
	4	FOR 5′- CGTATCGCCTCCCTCGCGCCATCAGAGCACTGTA GAATCCAGGTTGCCGTCAA -3′
		REV 5′- CTATGCGCCTTGCCAGCCCGCTCAGAGCACTGTAGATTCAATTTCATCTTCAGAGTGA -3′
4	1	FOR 5′- CGTATCGCCTCCCTCGCGCCATCAGACGAGTGCGTGGTTCAAGAGAAGTTCAGATAC -3′
		REV 5′- CTATGCGCCTTGCCAGCCCGCTCAGACGAGTGCGTTAATGGTGTAGATGCCTTCA -3′
	2	FOR 5′- CGTATCGCCTCCCTCGCGCCATCAGACGCTCGACAGGTTCAAGAGAAGTTCAGATAC -3′
		REV 5′- CTATGCGCCTTGCCAGCCCGCTCAGACGCTCGACATAATGGTGTAGATGCCTTCA -3′
	4	FOR 5′- CGTATCGCCTCCCTCGCGCCATCAGAGCACTGTAGGGTTCAAGAGAAGTTCAGATAC -3′
		REV 5′- CTATGCGCCTTGCCAGCCCGCTCAGAGCACTGTAGTAATGGTGTAGATGCCTTCA -3′
5	2	FOR 5′- CGTATCGCCTCCCTCGCGCCATCAGACGCTCGACACATGAGTGATTCCAACTATGTT -3′
		REV 5′- CTATGCGCCTTGCCAGCCCGCTCAGACGCTCGACACTGATACATCGCTTCTTCTG -3′
	4	FOR 5′- CGTATCGCCTCCCTCGCGCCATCAGAGCACTGTAGCATGAGTGATTCCAACTATGTT -3′
		REV 5′- CTATGCGCCTTGCCAGCCCGCTCAGAGCACTGTAGCTGATACATCGCTTCTTCTG -3′
	5	FOR 5′- CGTATCGCCTCCCTCGCGCCATCAGATCAGACACGCATGAGTGATTCCAACTATGTT -3′
		REV 5′- CTATGCGCCTTGCCAGCCCGCTCAGATCAGACACGCTGATACATCGCTTCTTCTG -3′

The primers cover exons 11–24 of the *FLT3* gene. Each forward and reverse primer consists of an adapter sequence for emulsion PCR, a sample specific barcode sequence (multiplex identifier) that allows sample pooling and a gene-specific sequence.

## References

[1] Levis M, Small D (2003). FLT3: ITDoes matter in leukemia. Leukemia.

[2] Schnittiger S, Scoch C, Dugas M, Kern W, Staib P, Wuchter C, Loffler H, Sauerland CM, Serve H, Büchner T, Haferlach T, Hiddemann W (2002). Analysis of FLT3 lenght mutations in 1003 patients with acute myeloid leukemia: correlation to cytogenetics, FAB subtype and prognosis in the AMLCG study and usefulness as a marker for the detection of minimal residual disease. Blood.

[3] Levis M (2013). FLT3 mutations in acute myeloid leukaemia: what is the best approach in 2013?. American Society of Hematology Education Program.

[4] Thiede C, Steudel C, Mohr B, Schaich M, Schächel U, Platzbecker U, Wermke M, Bornhäuser M, Ritter M, Neubauer A, Ehninger G, Illmer T (2002). Analysis of FLT3-activating mutations in 979 patients with acute myelogenous leukemia: association with FAB subtypes and identification of subgroups with poor prognosis. Blood.

[5] Marcucci G, Haferlach T, Döhner H (2011). Molecular genetics of adult acute myeloid leukemia: prognostic and therapeutic implications. Journal of Clinical Oncology.

[6] Cornelissen JJ, Gratwohl A, Schlenk RF, Sierra J, Bornhäuser M, Juliusson G, Råcil Z, Rowe JM, Russell N, Mohty M, Löwenberg B, Sociè G, Niederwieser D, Ossenkoppele GJ (2012). The European LeukemiaNet AML Working Party consensus statement on allogenic HSCT for patients with AML in remission: an integrated-risk adapted approach. Nature Reviews. Clinical Oncology.

[7] Schnittiger S, Bacher U, Kern W, Alpermann T, Haferlach C, Haferlach T (2011). Prognostic impact of FLT3-ITD load on NPM1 mutated acute myeloid leukemia. Leukemia.

[8] Gale RE, Green C, Allen C, Mead AJ, Burnett AK, Hills RK, Linch DC (2008). Medical Research Council Adult Leukaemia Working Party. The Impact of FLT3 internal tandem duplication mutant level, number, size, and interaction with NPM1 mutations in a large cohort of young adult patients with acute myeloid leukaemia. Blood.

[9] Linch DC, Hills RK, Burnett AK, Khwaja A, Gale RE (2014). Impact of FLT3 ITD mutant allele level on relapse risk in intermediate-risk acute myeloid leukaemia. Blood.

[10] Warren M, Luthra R, Yin CC, Ravandi F, Cortes JE, Kantarjian HM, Medeiros LJ, Zuo Z (2012). Clinical impact of change of FLT3 mutation status in acute myeloid leukemia patients. Modern Pathology.

[11] Cloos J, Goemans BF, Hess CJ, van Oostveen JW, Waisfisz Q, Corthals S, de Lange D, Boeckx N, Hählen K, Reinhardt D, Creutzig U, Schuurhuis GJ, Zwaan ChM, Kaspers GJ (2006). Stability and prognostic influence of FLT3 mutations in paired initial and relapsed AML samples. Leukemia.

[12] Welch JS, Ley TJ, Link DC, Miller CA, Lareson De, Koboldt DC, Wartman LD, Lamprecht TL, Liu F, Xia J, Kandoth C, Fulton RS, McLellan MD (2012). The origin and evolution of mutations in acute myeloid leukaemia. Cell.

[13] Krönke J, Bullinger L, Teleanu V, Tschürtz F, Gaidzik VI, Kühn MW, Rücker FG, Holzmann K, Paschka P, Kapp-Schwörer S, Späth D, Kindler T, Schittenhelm M (2013). Clonal evolution in relapsed NPM1-mutated acute myeloid leukemia. Blood.

[14] Metzker ML (2010). Sequencing technologies-the next generation. Nature Reviews Genetics.

[15] Kohlmann A, Klein HU, Weissmann S, Bresolin S, Chaplin T, Cuppens H, Haschke-Becher E, Garicochea B, Grossmann V, Hanczaruk B, Hebestreit K, Gabriel C, Iacobucci I (2011). The Interlaboratory RObustness of Next-generation sequencing (IRON) study: a deep sequencing investigation of TET2, CBL and KRAS mutations by an international consortium involving 10 laboratories. Leukemia.

[16] Grossmann V, Roller A, Klein HU, Weissmann S, Kern W, Haferlach C, Dugas M, Haferlach T, Schnittiger S, Kohlmann A (2013). Robustness of amplicon deep sequencing underlies its utility in clinical applications. Journal of Molecular Diagnostics.

[17] Soverini S, De Benedittis C, Machova Polakova K, Brouckova A, Horner D, Iacono M, Castagnetti F, Gugliotta G, Palandri F, Papayannidis C, Iacobucci I, Venturi C, Bochicchio MT (2013). Unraveling the complexity of tyrosine kinase inhibitor-resistant populations by ultra-deep sequencing of the BCR-ABL kinase domain. Blood.

[18] Bianchini M, Ottaviani E, Grafone T, Giannini B, Soverini S, Terragna C, Amabile M, Piccaluga PP, Malagola M, Rondoni M, Bosi C, Baccarani M, Martinelli G (2003). Rapid detection of FLT3 mutations in acute myeloid leukemia patients by denaturing HPLC. Clinical Chemistry.

[19] Stirewalt DL, Pogosova-Agadjanyan EL, Tsuchiya K, Joaquin J, Meschinchi S (2014). Copy-neutral loss of heterozygosity is prevalent and a late event in the pathogenesis of FLT3/ITD AML. Blood Cancer.

[20] Alvarado Y, Kantarjian HM, Luthra R, Ravandi F, Borthakur G, Garcia-Manero G, Konopleva M, Estrov Z, Andreeff M, Cortes JE (2014). Treatment with FLT3 inhibitor in patients with FLT3-mutated Acute Myeloid Leukemia is associated with development of secondary FLT3-tyrosine kinase domain mutations. Cancer.

[21] Wander SA, Levis MJ, Fathi AT (2014). The evolving role of FLT3 inhibitors in acute myeloid leukemia: quizartinib and beyond. Therapeutic Advantages in Hematology.

[22] Schlenk RF, Kayser S, Bullinger L, Kobbe G, Casper J, Ringhoffer M, Held G, Brossart P, Lübbert M, Salih HM, Kindler T, Horst HA, Wulf G (2014). Differential impact of allelic ratio and insertion site in FLT3-ITD-positive AML with respect to allogeneic transplantation. Blood.

[23] Döhner H, Estey EH, Amadori S, Appelbaum FR, Büchner T, Burnett AK, Dombret H, Fenaux P, Grimwade D, Larson RA, Lo-Coco F, Naoe T, Niederwieser D (2010). Diagnosis and management of acute myeloid leukemia in adults: recommendations from an international expert panel, on behalf of the European LeukemiaNet. Blood.

[24] Ding L, Ley TJ, Larson DE, Miller CA, Koboldt DC, Welch JS, Ritchey JK, Young MA, Lamprecht T, McLellal MD, McMichael JF, Wallis JW, Lu C (2012). Clonal evolution in relapsed acute myeloid leukemia revealed by whole genome sequencing. Nature.

[25] Ottone T, Zaza S, Divona M, Hasan SK, Lavorgna S, Laterza S, Cicconi L, Panetta P, Di Giandomenico J, Cittadini M, Ciardi C, Montefusco E, Franchi A (2013). Identification of emerging FLT3 ITD-positive clones during clinical remission and kinetics of disease relapse in acute myeloid leukaemia with mutated nucleophosmin. British Journal of Haematology.

[26] Guieze R, Cornillet-Lefebvre P, Lioure B, Blanchet O, Pigneux A, Recher C, Bonmati C, Fequeux N, Bulabois CE, Bouscary D, Vey N, Delain M, Turlure P (2012). Role of autologous hematopoietic stem cell transplantation according to the NPM1/FLT3-ITD molecular status for cytogenetically normal AML patients: a GOELAMS study. American Journal of Hematology.

[27] Thol F, Kölking B, Damm F, Reinhardt K, Klusmann JH, Reinhardt D, von Neuhoff N, Brugman MH, Schlegelberger B, Suerbaum S, Krauter J, Ganser A, Heuser M (2012). Next-generation sequencing for minimal residual disease monitoring in Acute Myeloid Leukemia patients with FLT3-ITD or NPM1 mutations. Genes, Chromosomes and Cancer.

[28] Kurosu T, Nagao T, Wu N, Oshikawa G, Miura O (2013). Inhibition of the PI3K/Akt/GSK3 pathway downstram of BCR/ABL, Jak2-V617F, or FLT3-ITD downregulates DNA damage-induced Chk1 activation as well as G2/m arrest and prominently enhances induction of apoptosis. PLoS One.

[29] Pemmaraju N, Kantarjian H, Andreeff M, Cortes J, Ravandi F (2014). Investigational FMS-like tyrosine kinase 3 inhibitors in treatment of acute myeloid leukaemia. Expert Opinion on Investigational Drugs.

[30] Ishikawa Y, Kiyoi H, Tsujimura A, Miyawaki S, Miyazaki Y, Kuriyama K, Tomonaga M, Naoe T (2009). Comprehensive analysis of cooperative gene mutations between class I and class II in de novo acute myeloid leukaemia. European Journal of Haematology.

[31] Baker SD, Zimmermann EI, Wang Y-O, Orwick S, Zatechka DS, Buaboonnam J, Neale GA, Olsen SR, Enemark EJ, Shurtleff S, Rubnitz JE, Mullighan CG, Inaba H (2013). Emergence of polyclonal FLT3 tyrosine kinase domain mutations during sequential therapy with Sorafenib and Sunitinib in FLT3-ITD-positive Acute Myeloid Leukemia. Clinical Cancer Research.

[32] Kohlmann A, Bacher U, Schnittiger S, Haferlach T (2014). Perspective on how to approach molecular diagnostics in acute myeloid leukemia and myelodysplastic syndromes in the era of next-generation sequencing. Leukemia and lymphoma.

[33] Haffer LG, McGowan-Jordan J, Schmid M (2013). An International System for Human Cytogenetic Nomenclature. Karger Publishers.

